# Adjusting for sampling variability in sparse data: geostatistical approaches to disease mapping

**DOI:** 10.1186/1476-072X-10-54

**Published:** 2011-10-06

**Authors:** Kristen H Hampton, Marc L Serre, Dionne C Gesink, Christopher D Pilcher, William C Miller

**Affiliations:** 1 Department of Epidemiology, University of North Carolina at Chapel Hill, Chapel Hill, NC, USA; 2 Department of Environmental Sciences and Engineering, University of North Carolina at Chapel Hill, Chapel Hill, NC, USA; 3 Dalla Lana School of Public Health, University of Toronto, Toronto, Ontario, Canada; 4 HIV/AIDS Division, San Francisco General Hospital, University of California-San Francisco, San Francisco, CA, USA; 5 Department of Medicine, University of North Carolina at Chapel Hill, Chapel Hill, NC, USA

**Keywords:** disease mapping, sampling variability, spatial distribution, spatial analysis, epidemiological methods

## Abstract

**Background:**

Disease maps of crude rates from routinely collected health data indexed at a small geographical resolution pose specific statistical problems due to the sparse nature of the data. Spatial smoothers allow areas to borrow strength from neighboring regions to produce a more stable estimate of the areal value. Geostatistical smoothers are able to quantify the uncertainty in smoothed rate estimates without a high computational burden. In this paper, we introduce a uniform model extension of Bayesian Maximum Entropy (UMBME) and compare its performance to that of Poisson kriging in measures of smoothing strength and estimation accuracy as applied to simulated data and the real data example of HIV infection in North Carolina. The aim is to produce more reliable maps of disease rates in small areas to improve identification of spatial trends at the local level.

**Results:**

In all data environments, Poisson kriging exhibited greater smoothing strength than UMBME. With the simulated data where the true latent rate of infection was known, Poisson kriging resulted in greater estimation accuracy with data that displayed low spatial autocorrelation, while UMBME provided more accurate estimators with data that displayed higher spatial autocorrelation. With the HIV data, UMBME performed slightly better than Poisson kriging in cross-validatory predictive checks, with both models performing better than the observed data model with no smoothing.

**Conclusions:**

Smoothing methods have different advantages depending upon both internal model assumptions that affect smoothing strength and external data environments, such as spatial correlation of the observed data. Further model comparisons in different data environments are required to provide public health practitioners with guidelines needed in choosing the most appropriate smoothing method for their particular health dataset.

## Background

Disease maps that summarize the spatial and spatio-temporal variation in rates of disease have a wide range of applications, from hypothesis generation to public health surveillance. Identification of areas with unusually high or low rates may indicate clusters or 'hot spots' of disease that can aid decisions regarding intervention or prevention programs, allocation of health care resources, or provide context for further epidemiological studies [1-4]. At the same time, the utility of disease mapping often depends on how accurately the value being mapped estimates the spatial process of interest. Estimating disease risk requires a defined, closed population and cannot be measured directly with surveillance data; accordingly, incidence rates are often used to approximate disease risk [5]. However, calculating crude rates from routinely collected health data indexed at a small geographical resolution poses specific statistical problems due to the sparse nature of the data, especially for rare diseases [1-6]. In particular, crude rate maps of small areas will be dominated by sampling variability. Due to variation in population size among areas, a map displaying crude rates will tend to be dominated by areas with small populations since small changes to the observed number of cases will result in large changes to the rate [5-7]. Meanwhile, observed high rates based on small populations are likely to be artificially elevated due to the high variability in the estimates. In other words, error due to sampling variability introduces observational noise into the map that may obscure the underlying spatial process of interest, and, if not adjusted for, lead to incorrect inference about the spatial pattern of interest.

One general method to improve the stability of observed rates is to increase population sizes by upscaling from finer to coarser resolution levels, such as from census tracts or ZIP codes to counties. Increasing the aggregation area, however, causes a loss in the resolution of the data, thereby masking spatial details needed to identify, analyze, and monitor health problems at the community level [5,7,8]. At the same time, analyzing health data at too fine a resolution, such as with case event or point maps, can compromise patient confidentiality and may be misleading in some circumstances because the underlying population density is not considered [8]. This paper focuses on the issue of sampling variability in disease maps at a geographical resolution corresponding to an aggregation of counts over *fixed small *geographical regions - in this case, postal codes of residence (US postal service 'ZIP codes').

Both deterministic and model-based approaches have been proposed to address the issue of sampling variability, also referred to as the 'small number problem,' when mapping health data at fine resolutions [1,5,9]. These approaches reduce the noise in rates of small areas through 'spatial smoothing,' which allows areas to borrow strength from neighboring regions to produce a more stable estimate of the value associated with each region [2-4,6,7,10,11]. Among the different methods, however, a tradeoff exists between the computational complexity of each method and the ability to account for spatial correlation and uncertainty in the smoothed results [12]. For example, methods such as disk smoothing, population-weighted averages, and empirical Bayes estimates have low computing requirements but do not account for spatial autocorrelation or quantify the amount of uncertainty in the smoothed rates [12,13]. In contrast, full Bayesian hierarchical models are able to incorporate multiple covariates in the model parameters to yield the full posterior distribution of the estimated rate, yet are computationally cumbersome due to time-consuming iterative procedures and challenging for non-statisticians to implement and interpret due to their complexity [2,12,13].

Geostatistical methods modified to account for the heteroscedasticity of health data, namely that the variance at each location varies as a function of the population size, offer a compromise between computational burden and quantification of the uncertainty in the smoothed rate estimate. Methods such as Poisson kriging are able to account for spatial correlation and yield a full posterior distribution while being computationally faster than fully Bayesian hierarchical models [12-14]. Furthermore, simulation studies of cancer mortality showed that Poisson kriging outperformed simple population-weighted averages, empirical Bayes smoothers, and the Besag-York-Mollié Bayesian hierarchical model in measures of estimation accuracy and degree of smoothing, particularly when background rate values were spatially correlated [12,13]. While spatial smoothing is needed to improve the stability of observed rates based on small populations, too much smoothing reduces the ability to identify areas of high or low rates that may indicate clusters or outbreaks of disease.

In this paper, we examine extensions of two geostatistical methods, ordinary kriging and Bayesian Maximum Entropy (BME), which have been used extensively in the analysis of fields exhibiting spatial variability and are computationally efficient [15-22]. Traditional geostatistics do not account for the heteroscedasticity of disease rates and must be modified to address both the numerator and denominator of rate data [12-14]. Poisson kriging as an extension of ordinary kriging was first introduced by Monestiez et al. [23] and applied to health data by Goovaerts [12]. Here, we introduce a uniform model extension of BME (UMBME) that, like Poisson kriging, can account for changes in variance due to population size and compare the performance of UMBME to that of Poisson kriging in measures of estimation accuracy and smoothing strength. We first examine differences in underlying model assumptions by applying each model to simulated datasets where the true spatial variation in the data is known. We then apply both models to the real data example of human immunodeficiency virus (HIV) infection with a spatial analysis of infection occurring in North Carolina HIV testing data from 1994 to 2002. Attempts to describe or monitor the distribution of HIV cases detected through testing have been limited by the variability in sampling that is inherent in the testing situation.

## Methods

### Model definitions

In traditional epidemiology, the incidence rate of disease is defined as the number of new cases in the at-risk population divided by the person-time, or summation of each person's observation time, over a specified period [5,24]. With rare diseases at steady state, the loss of person-time per diseased person is minimal, and the total person-time at risk may be approximated by the total size of the at-risk population over the period times the length of the period (day, month, year) [5,24]. In other words, the incidence rate corresponds to a proportion of new cases in the at-risk population divided by the time duration considered to enumerate the new cases.

Following this concept, we define in the spatial context the *latent *incidence rate as the theoretically possible rate of infection built on a hypothetical infinite underlying population at risk. In this paper, we consider observation datasets aggregated to a single time period *t*, resulting in space only analyses and temporally independent maps that can be used to examine the spatial variability of the random fields. Therefore, without loss of generality, the latent incidence rate of disease in a given region *i *can be defined as:

(1) $$X_{i}=\underset{n_{t}\to \infty }{\lim}\frac{Y_{i}}{n_{i}}\;,\;\;\;i=1,...I\;$$

where *X_i _*is the latent disease rate, *Y_i _*is the number of new cases of disease, and *n_i _*is the size of the population at risk in area *i*. In other words, as the size of the population *n_i _*reaches infinity, then the observed disease rate *Y_i_*/*n_i _*approaches the latent disease rate for that location. In this hypothetical situation, sampling variability is minimized because all regions being mapped are of equally large populations and small changes in the number of cases do not result in large changes to the observed rate.

In practice, however, only a finite population at risk *n_i _*in area *i *may be measured, producing a finite number of cases *Y_i _*and an observed rate *R_i _*= *Y_i_*/*n_i_*. Note that even when *n_i _*is an exhaustive count of the entire population at risk in area *i*, maps of the observed rate *R_i _*are subject to noise due to variation in population size among regions, and areas with rates based on small populations will tend to dominate the map. The difference between the observed rate *R_i _*and latent rate *X_i _*is directly related to the observation that *R_i _*can only be defined in increments of 1/*n_i_*, while the underlying latent rate can take any value on the real number continuum. The difference between *R_i _*and *X_i _*is inherently a measurement error *ε_i _*that can be expressed as:

(2) $$R_{i}=X_{i}+\varepsilon_{i}$$

where we refer to *ε_i _*as the error due to sampling variability in area *i*. In this paper, we contrast two approaches in modeling sampling variability, Poisson kriging and the UMBME method, which use unbounded and bounded distributions, respectively, for the error *ε_i_*.

### Poisson kriging

With Poisson kriging, the observed number of cases is modeled as a random variable *Y_i _*that follows a Poisson distribution with one parameter. This parameter is the expected number of cases defined as the product of the population size *n_i _*by the latent rate *X_i_*, such that:

(3) $$Y_{i}|X_{i}\sim \text{Poisson}\left(n_{i}X_{i}\right)$$

As detailed by Goovaerts [12], the Poisson kriging latent rate in area *i *is estimated as a linear combination of *K *neighboring observed rates, such that:

(4) $${\hat{X}}_{i}^{PK}=\overset{K}{\underset{j=1}{\sum }}\lambda_{ij}R_{j}$$

where the weights *λ_ij _*are derived from a Poisson kriging specific system of linear equations, in which the sampling variability at *i *is accounted for by a normally distributed error *ε_i _*with variance equal to *σ*_*i*_^2 ^= m*/*n_i_*, where m* is the population-weighted mean of the observed rates [12]. We implemented the Poisson kriging adaptation of ordinary kriging in the MATLAB programming environment [25]. Detailed descriptions of Poisson kriging may be found in Goovaerts (2005) and Goovaerts and Gebreab (2008).

### Uniform model extension of Bayesian Maximum Entropy (UMBME)

An underlying assumption of UMBME is that the observed population *n_i _*is a representative sample of the population at risk in area *i *and measured without error. In this case, the observed number of cases *Y_i _*given the latent rate *X_i _*can be interpreted as the product of the population size and latent rate rounded to the nearest integer, such that:

(5) $$Y_{i}|X_{i}=\text{round}\left(n_{i}X_{i}\right)$$

The discrete nature of case counts requires *Y_i _*to be a whole number value. Therefore, when given an observed case count *Y_i _*and population *n_i_*, the latent rate *X_i _*follows a uniform distribution bounded by the rounding error, such that:

(6) $$X_{i}|R_{i}=U\left(R_{i}-\frac{0.5}{n_{i}},R_{i}+\frac{0.5}{n_{i}}\right)$$

where *R_i _*is the observed rate *Y_i _*/*n_i_*. In other words, in UMBME the measurement error *ε_i _*is assumed to be uniformly distributed in an interval of size 1/*n_i_*, which expresses the fact that *R_i _*is interpreted as an observation of the latent rate *X_i _*in increments of 1/*n_i_*. To derive the UMBME latent rate estimate from observed values, the BME method treats the observed values as 'soft data' with measures of uncertainty as defined by the uniform distribution. As detailed by Christakos et al. [17], the BME method processes information known about the rate field in three main stages: (i) Structural (or prior) stage, (ii) Specificatory (or meta-prior) stage, and (iii) Integration stage. Programs to perform each stage were derived from functions found in the BMElib software package for the MATLAB programming environment [25,26]. Detailed descriptions of the BME method may be found in Christakos and Li (1998) and Christakos et al. (2002), among others. A general introduction to information processing in BME is presented in Supplementary Materials, Appendix A.

In this study, the UMBME estimate ${\hat{X}}_{i}^{UMBME}$ of the latent rate in area *i *is the expected value of the posterior probability density function (pdf) derived in the Integration stage. Other possible estimators include the mode or median of the posterior pdf. Furthermore, the variance of the posterior pdf, or BME variance, provides a useful measure of the estimation uncertainty.

### Neighborhood selection

Poisson kriging and UMBME offer at least two advantages in neighborhood selection over their counterparts, particularly simple smoothers that weight all neighbors falling within an arbitrary fixed distance of areal centroids. First, both methods have the ability to limit the number of neighbors selected to a user-defined maximum, which can account for changes in the spatial density of centroids, and thus the size of geographical units, across the study area [12,17]. For example, an urban area may have dozens of neighbors falling within a fixed distance while a rural area may only have a handful of neighbors falling within the same distance. Without limitations on the number of selected neighbors, the urban area would have significantly more contributors to its smoothed value than the rural area. Second, Poisson kriging and UMBME are able to determine smoothing weights based upon the data's covariance model. If the neighborhood distance selected is large enough such that it includes all the neighborhood information, then increasing the distance or number of points does not add more information or change the model estimation.

### Simulated data analysis

**Figure 1:**
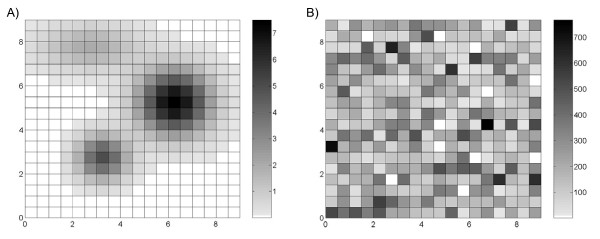
**Maps of the simulated A) latent rate field *X(s) *and B) sample population size *n(s)***. The values of *X(**s**) *and *n(**s**) *ranged from 0 to 7.5 per 1,000 persons and from 1 to 767 persons, respectively.

We applied both Poisson kriging and UMBME to simulated data where the true spatial variation in the latent rate field *X*(***s***) is known in order to examine the effect of underlying assumptions on each model's performance in measures of estimation accuracy and smoothing strength. Simulations allow us to both model the true latent disease rate and to control variables in order to explore how changes impact the smoothed result [12,13]. For example, we considered a study area divided into square regions with the spatial location ***s ***of each region defined as the center of the corresponding square (Figure 1), which minimized the effect of spatial support and the modifiable areal unit problem on the smoothed estimates since regions were of uniform size and shape [12]. With real data, not knowing the true latent rate or relative impact of error sources makes it difficult to assess the accuracy and validity of different smoothing methods.

Since sampling variability generates the greatest distortion in studies of rare diseases, a low latent rate field *X(**s**) *was simulated where the latent rate of disease in each region ranged in value from 0 to 7.5 per 1,000 persons (Figure 1A). We modeled the latent rate field by first identifying one region each of relative low, medium, and high latent rates with an associated decay rate. We then calculated the latent rates for surrounding areas based upon the distance from each central location. The resulting disease field where higher rates tend to be grouped around a central location follows the core area theory of sexually transmitted diseases such as syphilis [19,27].

The sample population size *n(**s**) *was then determined using a random number generator, resulting in values ranging from 1 to 767 (Figure 1B). While general population density tends to be spatially correlated, it is rare for the populations sampled by public health investigations and surveillance systems to be perfectly representative of the general population. Instead, factors such as outreach efforts and concentrations of special populations can cause the populations sampled in neighboring areas to vary significantly. Furthermore, population density is dependent on geographic scale. Even in urban study areas, the population density of, for example, census block groups may represent a random patchwork depending on the locations of commercial and residential areas.

**Figure 2:**
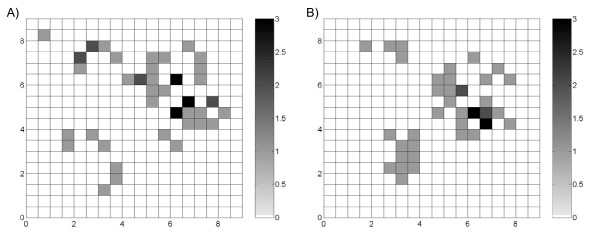
**Maps of the simulated case count *Y(s) *under the A) Poisson assumption and B) uniform assumption**. Under the Poisson assumption, the observed cases are drawn from a Poisson distribution. Under the uniform assumption, the observed cases are derived from the product of the latent rate and population size. In both realizations, the values of *Y(**s**) *ranged from 0 to 3 cases.

A fundamental difference in the model assumptions of Poisson kriging and UMBME is the probability distribution of the observed cases, *Y(**s**)*, given the latent rate, *X(**s**)*. If the observed cases, *Y(**s**)*, are drawn from a Poisson distribution, then Poisson kriging should outperform UMBME in estimating the latent rate. Conversely, if *Y(**s**) *integer values are derived from the product of the latent rate, *X(**s**)*, and population size, *n(**s**)*, then UMBME should outperform Poisson kriging in estimation accuracy. We derived two realizations of the case count, *Y(**s**)*, to verify these characteristics and examine differences in spatial variability between assumptions. Given the latent rate field, *X(**s**)*, and sample population, *n(**s**)*, one realization of *Y(**s**) *was sampled from a Poisson distribution (Figure 2A), while the other resulted from the product of *X(**s**) *and *n(**s**) *(Figure 2B). In both realizations, *Y(**s**) *ranged in value from 0 to 3 cases.

For a given realization, we have the simulated (true) *X*(***s***) and the observed rate, *R(**s**)*, from which we calculated both the Poisson kriging estimate, ${\hat{X}}_{i}^{PK}$, and UMBME estimate, ${\hat{X}}_{i}^{UMBME}$. We then calculated two statistics as measures of estimation accuracy: the mean square error (MSE) and Lin's Concordance Correlation Coefficient (LCCC) between the estimated value (${\hat{X}}_{i}^{PK}$ or ${\hat{X}}_{i}^{UMBME}$) and the simulated true value for *X(**s**)*. Divergence between the estimated and true latent rate values decreases as the MSE approaches zero and LCCC approaches one [28]. Meanwhile, smoothing strength was described as the degree to which each technique modified high or low observed rates towards the global mean. More smoothing indicates greater movement towards the global mean and less spatial variability in estimated values. While smoothing strength may be qualitatively assessed by examining, for example, whether regions of high observed rates are discernable in the estimated map, we also calculated the mean absolute difference (MAD) between estimated values and the mean of observed rates. As the MAD approaches zero, the average deviation of estimated values from the global mean decreases, indicating greater smoothing of observed values.

### Mapping HIV in North Carolina

To examine model performance in real world situations, we mapped HIV disease rates in North Carolina using both Poisson kriging and UMBME. Previous studies of sexually transmitted infections (STIs) demonstrate that cases tend to be concentrated in residential core areas with high rates of infection in small, definable geographic areas [19,21,27,29-31]. In North Carolina, approximately 1,700 new diagnoses of HIV disease are reported to the North Carolina public health surveillance system each year, with about 25 per cent of North Carolina's HIV disease reports consistently coming from rural, or non-metropolitan, areas since the early 1990s [32]. Regional differences in the spread of HIV highlight the necessity for accurate information on the spatial distribution of the disease in order to effectively target prevention activities and identify possible community-level risk factors contributing to the epidemic.

In this analysis, we calculated rates of new HIV disease reports in North Carolina's testing population from 1994 to 2002. Specifically, clients at risk of HIV infection presenting to publicly-funded Voluntary Counseling and Testing (VCT) sites distributed across the state of North Carolina were tested for long-term HIV infection. ZIP code of residence was recorded in a research database for each de-identified test result. Records for clients reporting previous positive HIV tests were excluded from analysis. The remaining records were geocoded to the residential-weighted centroid of the reported ZIP code of residence [33]. There were 938,889 total tests and 5,677 new HIV cases reported between 1994 and 2002, of which 96% to 97% matched to a location. Reasons for failing to geocode included having a missing, invalid, or out-of-state ZIP code. We modeled the observed HIV infection rate using Poisson kriging and UMBME and evaluated model performance.

In real world situations, the true latent rate of disease is unknown. Therefore, in order to assess model fit, we used cross-validatory predictive checks, which have been shown to be useful in determining whether divergent rates are due to poor model fit or to actual 'hot-spots' of disease that warrant further investigation [34,35]. The basic concept of cross-validation is to remove each observed rate in turn, in this case observed rates assigned to a ZIP code population centroid, then reanalyze the original data without that observation, and assess the model's ability to predict the removed area's data value. Estimation accuracy was then measured as the MSE and LCCC between the model-predicted value and the observed rate. Smoothing strength was assessed both qualitatively and as the MAD between model-predicted values and the mean of observed rates.

## Results and discussion

### Simulated data analysis

In applying smoothing models to the simulated rate field *R(**s**)*, we considered first the spatial moments of the latent rate spatial random field *X(**s**)*. In the absence of prior information regarding the general behavior of *X(**s**)*, we assigned no mean trend to the model and used the information provided by the rate field *R(**s**) *directly in our calculations rather than as a residual with the mean trend removed. This enabled us to model the spatial variability of *X(**s**) *using the covariance of *R(**s**)*. A useful feature of the covariance model is that it does not require prior distributional assumptions of the data and can be applied to both the Poisson and uniform simulations. Other estimators, such as Monestiez et al.'s population-weighted semivariogram and Yu et al.'s Poisson regression model in BME, have measurable advantages but are dependent on the Poisson assumption [23,36]. Furthermore, covariance plots provide a quantitative assessment of the correlation between pairs of points and are useful in assessing the strength and scale of the disease pattern [19]. For example, the covariance range, or distance where the curve becomes asymptotic to the x-axis, may be used to identify the neighborhood of influence around an observation point. Observations within the covariance range may influence values at the current location, whereas observations outside this range may not be influential. In addition, local disease patterns may be described by the behavior of the covariance model near the origin. Steep curves indicate rapid change over a short distance, while long-tailed curves indicate slower change and less variability over the same distance.

### Poisson assumption

**Figure 3:**
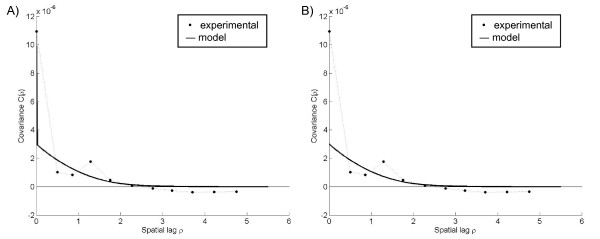
**Plots of the experimental rate covariance with covariance models under the Poisson assumption**. The nugget component of the A) observed rate field *R(**s**)*-covariance model is due to sampling variability error and removed to obtain the B) *X(**s**)*-covariance model.

The experimental covariance calculated from the corresponding observed rates shows the change in the correlation between pairs of points as the distance between points increase in space. For the Poisson assumption, the experimental covariance indicated high local variability with a neighborhood of influence extending less than 2 spatial units from each point (Figure 3). The corresponding *R(**s**)-*covariance model (Figure 3A) was obtained by fitting a nugget-exponential-Gaussian nested model to the experimental *R(**s**)-*covariance values, such that

(7) cRρ=c1δρ+c2 exp-3ρaρ1+c3 exp-3ρ2aρ22

where *ρ *is the spatial lag, *c*_1_, *c*_2_, and *c*_3 _are constants whose sum equals the variance of *R(**s**)*, *δ_ρ _*is the Kronecker delta function, and *a_ρ1 _*and *a_ρ2 _*represent the spatial ranges of the exponential and Gaussian components, respectively. The nugget component of this covariance model corresponds to the initial drop of the covariance, while the exponential and Gaussian components correspond to the tail of the covariance curve. This covariance model is adequate if we are interested in mapping the observed rate field *R(**s**) *over the geographical region of interest using observed rate data. On the other hand, if we are interested in mapping the true latent rate field *X(**s**)*, we then need to model its covariance function. We obtain the *X(**s**)-*covariance model by simply recognizing that the nugget component (initial drop of covariance) of the *R(**s**)*-covariance is due to the sampling variability error term *ε *defined in 2. Therefore, we obtained the *X(**s**)-*covariance model (Figure 3B) by removing the nugget component of the *R(**s**)-*covariance model and keeping only the exponential and Gaussian components, such that

(8) cXρ=c2 exp-3ρaρ1+c3 exp-3ρ2aρ22

where *ρ *is the spatial lag, *c*_2_, and *c*_3 _are constants, and *a_ρ1 _*and *a_ρ2 _*represent the spatial ranges of the exponential and Gaussian components, respectively.

**Figure 4:**
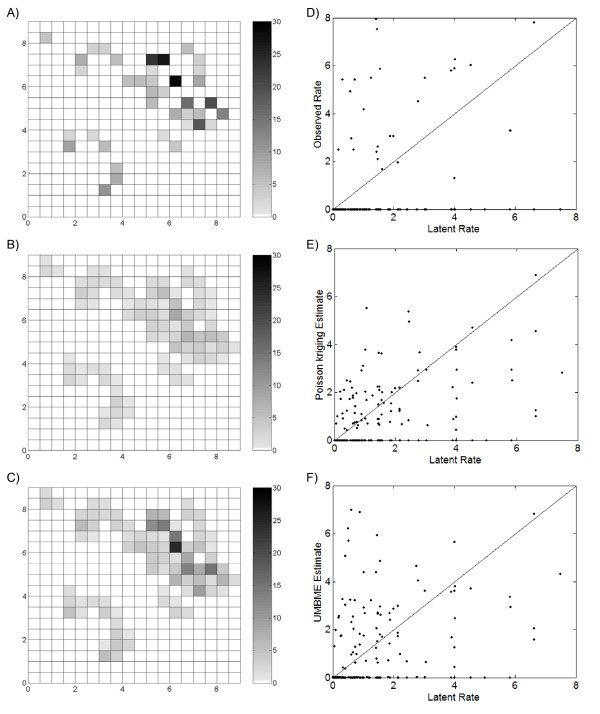
**Maps and scatterplots of the observed, Poisson kriging estimated, and UMBME estimated rates under the Poisson assumption**. Compared with the A) observed rate map, the B) Poisson kriging map displayed greater smoothing than the C) UMBME map, particularly in areas of high observed rates (cases per 1,000 persons). Meanwhile, scatterplots of the D) observed, E) Poisson kriging estimated, and F) UMBME estimated rates versus the true latent rate, *X(**s**)*, combined with MSE and LCCC calculations (Table 1), demonstrated that Poisson kriging produced the highest estimation accuracy under the Poisson assumption.

The observed rates *R_i_*, calculated from the cases simulated under the Poisson kriging assumption, ranged in value from 0 to 29.1 per 1,000 persons (Figure 4A). Using these observed rates and the corresponding covariance models (Figure 3), we obtained the Poisson kriging estimate ${\hat{X}}_{i}^{PK}$ (Figure 4B) and the UMBME estimate ${\hat{X}}_{i}^{UMBME}$ (Figure 4C). Spatially, ${\hat{X}}_{i}^{PK}$ and ${\hat{X}}_{i}^{UMBME}$ exhibited nearly identical spatial distributions of non-zero values. This result is due in part to the similar identification of neighbors and integration of spatial correlation between the models. However, when compared with the observed rate map, Poisson kriging exhibited greater smoothing strength than UMBME, particularly in areas of high observed rates, such as region (6,6) of the study domain. Similarly, the Poisson kriging MAD was lower than that of UMBME (Table 1), indicating greater smoothing towards the observed mean with Poisson kriging than UMBME. With regards to estimation accuracy, scatterplots of the observed rates *R_i_*, the Poisson kriging estimates ${\hat{X}}_{i}^{PK}$, and the UMBME estimates ${\hat{X}}_{i}^{UMBME}$ versus the true latent rates *X_i _*(Figures 4D, E, and 4F, respectively) show that the closer a point is to the 45 degree best fit line, the more accurately the observed or estimated value approximates the true *X*(***s***)-value. Artificially elevated rates fall above the best fit line, with the observed rate plot (Figure 4D) showing the greatest deviation from the true *X*(***s***)-value. Quantitatively, Poisson kriging exhibited the highest estimation accuracy in measures of both MSE and LCCC when compared with the true *X*(***s***)-value (Table 1).

**Table 1:** Measures of model estimation accuracy, smoothing strength, and variance quality for the simulated Poisson assumption data, simulated uniform assumption data, and real HIV data.

Method	MSE	LCCC	MAD	*G*
Poisson Simulation				
Observed	1.00E-05	0.214		
Poisson kriging	**1.47E-06**	**0.550**	**0.938**	0.669
UMBME	4.83E-06	0.396	1.33	**0.798**
				
Uniform Simulation				
Observed	1.52E-06	0.618		
Poisson kriging	1.15E-06	0.555	**0.510**	0.670
UMBME	**6.43E-07**	**0.794**	0.701	**0.781**
				
HIV Data				
Observed	1.87E-04	0.013		
Poisson kriging	1.80E-04	0.039	**0.00297**	
UMBME	**1.79E-04**	**0.040**	0.00369	

The MSE and LCCC for the simulated data describe divergence between the model estimated and true latent rate values. For the HIV data, the MSE and LCCC values describe divergence between the model cross-validation results and observed rate values. Bolded values represent the model with the greatest estimation accuracy (MSE, LCCC), greatest smoothing strength (MAD), or best fit variance (*G*) in each dataset.

### Uniform assumption

**Figure 5:**
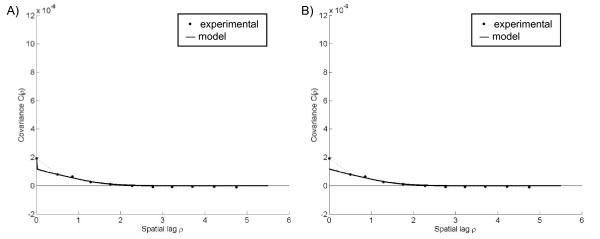
**Plots of the experimental rate covariance with covariance models under the uniform assumption**. The nugget component of the A) observed rate field *R(**s**)*-covariance model was removed to obtain the B) *X(**s**)*-covariance model.

Under the uniform assumption, the observed cases, *Y(**s**)*, were derived from the product of the latent rate field, *X*(***s***), and sample population, *n(**s**) *(Figure 2B). We then obtained experimental and model covariance values for the corresponding observed rate field *R(**s**) *(Figure 5A) and latent rate field *X(**s**) *(Figure 5B). Similar to those of the Poisson assumption, the experimental covariance indicated a neighborhood of influence extending less than 2 spatial units from each point. However, the experimental covariance under the uniform assumption had a smaller nugget effect, indicating lower local variability [5]. In other words, observations that were close in space had more similar values under the uniform assumption than the Poisson assumption.

**Figure 6:**
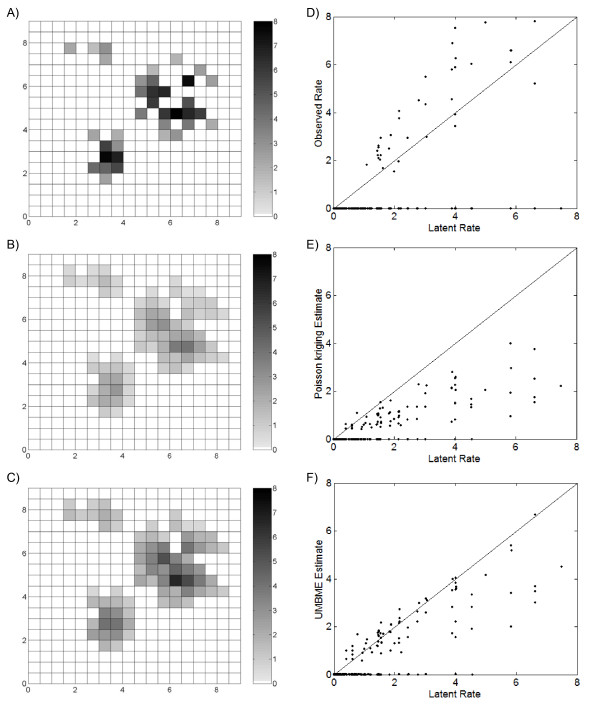
**Maps and scatterplots of the observed, Poisson kriging estimated, and UMBME estimated rates under the uniform assumption**. Compared with the A) observed rate map, the B) Poisson kriging map displayed greater smoothing than the C) UMBME map (cases per 1,000 persons). However, scatterplots of the D) observed, E) Poisson kriging estimated, and F) UMBME estimated rates versus the true latent rate, *X(**s**)*, combined with MSE and LCCC calculations (Table 1), demonstrated that UMBME produced the highest estimation accuracy under the uniform assumption.

The observed rates *R_i_*, calculated from the cases simulated under the uniform assumption, ranged in value from 0 to 7.5 per 1,000 persons (Figure 6A). Using these observed rates and the corresponding covariance models (Figure 5), we obtained the Poisson kriging estimates ${\hat{X}}_{i}^{PK}$ (Figure 6B) and the UMBME estimates ${\hat{X}}_{i}^{UMBME}$ (Figure 6C). Once again, the Poisson kriging and UMBME maps displayed nearly identical spatial distributions of non-zero values, while Poisson kriging exhibited greater smoothing strength than UMBME with both less discernible high rate areas, such as region (6,5), and a lower MAD value (Table 1). In this case, however, the additional smoothing provided by Poisson kriging resulted in greater disparity with the true *X*(***s***)-value than UMBME, with a greater proportion of UMBME estimated points falling along the 45 degree best fit scatterplot line than those of Poisson kriging (Figures 6D, E, and 6F). Quantitatively, UMBME exhibited the highest estimation accuracy in measures of both MSE and LCCC (Table 1).

### Mapping HIV infection in North Carolina

**Figure 7:**
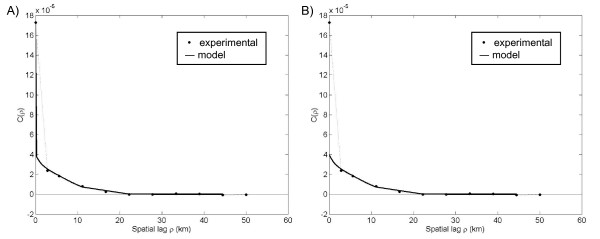
**Plots of the experimental rate covariance with covariance models for the North Carolina HIV data**. The experimental covariance indicated a spatial neighborhood of influence extending less than 20 kilometers from each location. The nugget component of the A) observed rate field *R(**s**)*-covariance model was removed to obtain the B) *X(**s**)*-covariance model.

**Figure 8:**
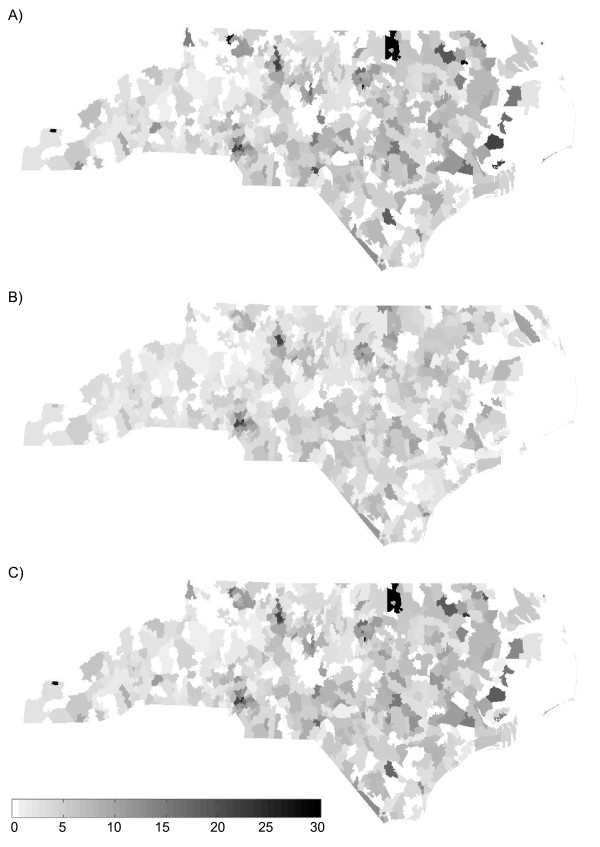
**Maps of the North Carolina HIV A) observed, B) Poisson kriging estimated, and C) UMBME estimated rates**. While the Poisson kriging and UMBME maps displayed nearly identical spatial distributions of non-zero values, Poisson kriging exhibited greater smoothing strength than UMBME, thereby providing less distinction between areas of high and low rates (cases per 1,000 tests).

We applied Poisson kriging and UMBME to North Carolina HIV disease data to examine their performance in a real-world situation. We derived experimental and model covariance values for the observed rate field (Figure 7A) and corresponding *X*(***s***) field (Figure 7B). The experimental covariance indicated a spatial neighborhood of influence extending less than 20 km from each location. While a nugget effect was present, without a point of comparison, it was difficult to ascertain whether the amount of spatial correlation was large or small for the given disease and geographical region. We then obtained maps of the HIV observed rate, the Poisson kriging estimates ${\hat{X}}_{i}^{PK}$, and the UMBME estimates ${\hat{X}}_{i}^{UMBME}$ (Figures 8A, B, and 8C, respectively). Similar to the simulated data analyses, Poisson kriging exhibited greater smoothing strength than UMBME with both less distinction between areas of high and low rates, such as in the northeast corner of the state, and a lower MAD value (Table 1). In cross-validation, UMBME performed only slightly better than Poisson kriging in MSE and LCCC values, with both models performing better than the observed data model with no smoothing (Table 1).

### Model comparison

Under both the Poisson and uniform assumptions of the simulated data, Poisson kriging exhibited greater smoothing strength than UMBME. The two methods utilize the same values for a number of parameters that affect smoothing strength, such as the maximum number of neighbors used in the estimation, the population size, and the covariance model [13]. The two methods differ, however, in their measurement error variances of the observed values and in their distributions of the posterior pdf. The error variance term is m*/*n_i _*for Poisson kriging, where m* is the population-weighted mean of the observed rates and *n_i _*is the population in area *i*, while the UMBME error term is distributed in an interval of size 1/*n_i_*. Furthermore, Poisson kriging yields a Gaussian posterior pdf characterized by the kriging estimate and variance, thereby allowing extreme values due to the tail of the Gaussian distribution [13]. UMBME, on the other hand, yields a non-Gaussian posterior pdf that is truncated by the interval soft data. Possible UMBME estimated values are therefore limited by the bounds of the truncated posterior pdf.

The distributional form of the posterior pdf may also contribute to differences in the estimated variance of each method. Under both the Poisson and uniform assumptions of the simulated data, the Poisson kriging and UMBME estimated variances were generally inversely proportional to the areal population size, while the magnitude of the Poisson kriging variance was consistently higher than that of UMBME. However, of greater interest is how well the estimated variance captures the true latent rate. Assuming normality of the prediction errors, we calculated each method's statistical coverage, or probability that the true latent rate in each area falls within the *p*-probability interval characterized by the estimated mean and variance. For example, for a *p *value of 0.95, the ideal model coverage probability would also equal 0.95. We then calculated Deutsch's "goodness" statistic [12,37] to examine divergence between the model-estimated and theoretical probability intervals, such that

(9) $$G=1-\frac{1}{L}\overset{L}{\underset{l=1}{\sum }}w\left(p_{l}\right)\;|\varsigma \left(p_{l}\right)-p_{l}|\;\text{with}\;0\leq G\leq 1$$

where *L *is the discretization level of the computation (i.e. *L *= 95 when *p_l _*= 0.95), ς(*p_l_*) is the model-estimated coverage for probability interval *p_l_*, and w(*p_l _*)= 1 if ς(*p_l_*)>*p_l_*, and 2 otherwise. Double weight is given to deviations where the model-estimated coverage is less than expected [12]. The goodness of fit of the estimated variance increases as the value of *G *approaches one. With *L *= 100, UMBME outperformed Poisson kriging in *G *values for both the Poisson and uniform simulations (Table 1).

While a drawback of Poisson kriging's greater smoothing strength is that it becomes harder to distinguish areas of high or low estimated rates, in measures of estimation accuracy Poisson kriging performed better than UMBME under the Poisson assumption, UMBME performed better than Poisson kriging under the uniform assumption, and both models performed better than the observed rate with no smoothing. The Poisson assumption experimental covariance had a greater nugget effect than that of the uniform assumption, indicating less spatial correlation among the Poisson-derived observed rates than the uniform-derived rates, as shown in Figures 3 and 5. These results support those of previous simulation studies, which show that methods with greater smoothing strength are more accurate estimators when the spatial autocorrelation of the observed data is low, while methods that smooth less have greater estimation accuracy with data that is more spatially correlated [12].

**Figure 9:**
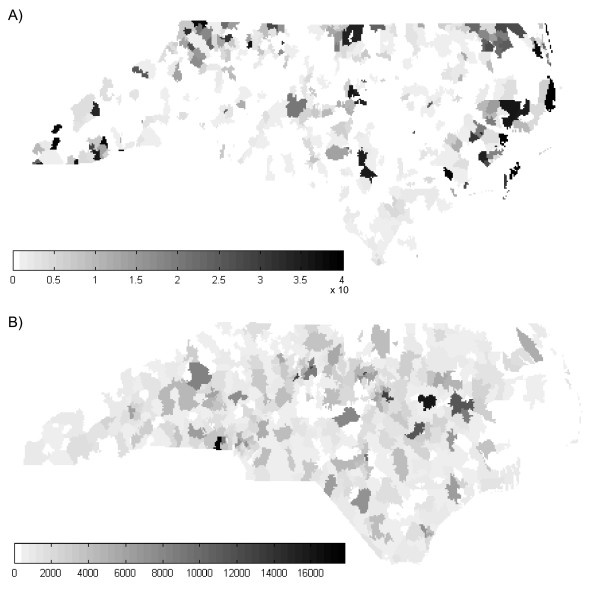
**Maps of the A) UMBME estimated variance and B) testing population for the North Carolina HIV dataset**. The estimated variance was generally inversely proportional to the underlying population size.

In a real-world situation where the true latent rate of disease is unknown, the Poisson kriging and UMBME estimated HIV rate maps compared with the observed HIV rate map (Figure 8) show how the noise generated by artificially high rates due to sampling variability may be reduced through smoothing. Smoothing out sampling variability is particularly important in the study of HIV infection in North Carolina because it facilitates correction of the rates observed in rural areas with small testing populations by borrowing strength from rates observed in neighboring areas with larger testing populations. By exhibiting greater smoothing strength, the Poisson kriging map (Figure 8B) also eliminated much of the distinction between high and low rates needed to identify outbreaks or clusters of disease. The UMBME map (Figure 8C), on the other hand, provided a similar spatial representation of HIV that smoothed out extreme rates based on small populations while maintaining areas of high observed rates that may be useful in monitoring the HIV epidemic. For example, the number of areas with rates above 10 cases per 1,000 tests was reduced by 59% with Poisson kriging, but only 12% with UMBME as compared with the observed rates (Table 2). Both Poisson kriging and UMBME, however, produced nearly identical cross-validation MSE and LCCC values. Without knowing the true latent rate of infection, additional information, such as the distributional form of the dataset, is needed to assess model fit. Similarly, we could not calculate Deutsch's goodness statistic, *G*, for the real data to assess model uncertainty because the true latent rate of infection was unknown. However, as with the simulated data, the model-estimated variances (Figure 9A for UMBME) were generally inversely proportional to the underlying population size (Figure 9B).

**Table 2:** Number of ZIP codes with values above the 90^th ^percentile of observed rates.

Method	No. ZIPs > 10 cases/1,000 tests	%Δ from Observed
Observed	74	--
UMBME	65	-12%
Poisson kriging	30	-59%

## Conclusions

Geostatistics modified to account for the specific nature of health data provides a rich class of methods able to incorporate and output more information about the disease field than simple smoothers while being computationally faster and easier to implement than full Bayesian hierarchical methods. This facilitates the investigation of both internal and external factors that affect model performance. As shown in this paper, both Poisson kriging and UMBME smoothing models more accurately predict the latent rate than the observed data with no smoothing. Meanwhile, Poisson kriging yielded smoother results than UMBME due to internal model assumptions.

Choosing the most appropriate smoothing method depends heavily on the characteristics of the disease being studied and the geographical space. As shown with the simulation data, accuracy of each estimation model was associated with the observed spatial correlation of the disease field. Methods that smoothed less performed better as the spatial correlation of the disease field increased. However, the observed spatial correlation, in turn, depends on the assumptions and characteristics of the latent and observed rate fields. For the latent rate, spatial characteristics of the disease, such as geographic risk factors and disease frequency, must be considered. For example, STIs tend to be concentrated in small, definable geographic areas where cases reside [19,21,27,29-31]. Therefore, maps of the residential locations of STI cases would typically exhibit greater spatial correlation than maps of the residential locations of a non-transmissible disease, such as leukemia. As a result, estimation methods with limited smoothing, such as UMBME, would be expected to produce more accurate predictions of the latent rate than methods that smooth more. However, the robustness of the observed data must also be considered. Factors that would increase or decrease the observed spatial correlation include the spatial support of the study area, such as the size and shape of geographical units, and the temporal resolution of the study. Observations measured over small time durations, such as monthly for STIs in North Carolina, tend to be spatially less correlated than observations identified over longer time periods, such as quarterly or annually.

Finally, in real world situations, the relative degree of spatial correlation in the observed data is difficult to ascertain when no point of reference exists. Further research is needed to compare the advantages and disadvantages of different smoothing models, examine model performance in different data environments, and examine model performance using different estimators. Additional research is also needed to examine whether fitting real data by a distributional form improves model performance and to identify other information about the disease field that would aid investigators in choosing the most appropriate smoothing model. Better disease map construction would improve the ability of public health officials to monitor spatial and temporal trends in disease rates, creating new opportunities for the definition of at-risk populations, identification of outbreaks, and allocation of resources toward areas and populations most affected by disease.

## List of Abbreviations

BME: Bayesian Maximum Entropy; HIV: Human Immunodeficiency Virus; LCCC: Lin's Concordance Correlation Coefficient; MAD: Mean Absolute Difference; MSE: Mean Square Error; Pdf: probability density function; STI: Sexually Transmitted Infection; UMBME: Uniform Model extension of Bayesian Maximum Entropy; VCT: Voluntary Counseling and Testing.

## Competing interests

The authors declare that they have no competing interests.

## Authors' contributions

KHH contributed to the study design, completed the analyses, and drafted the manuscript. MLS contributed to the study design, execution of the analyses, and interpretation of results. DCG, CDP, and WCM contributed to the study design and interpretation of results. All authors read and approved the final manuscript.

## Supplementary Materials

### APPENDIX A: The BME method

In the spatial application of BME, the distribution of a disease rate field is represented in terms of a Spatial Random Field (SRF), *X(**s**)*, where ***s ***is the spatial location. Each region *i *is defined in terms of a point spatial location, such as the latitude and longitude of the areal centroid, where ***s_i _***= (*s_1i _*, *s_2i_*) and *i *= 1,...*I*. The spatial moments of *X(**s**) *can be used to describe the behavior of the SRF. For example, the mean function

(A1) $$m_{x}\left(s\right)=\bar{X\left(s\right)}$$

(the overbar denotes stochastic expectation), characterizes trends and systematic structures in space, while the covariance function

(A2) $$c_{x}\left(\rho \right)=\bar{\left[X\left(\text{s}\right)-\bar{X}\left(\text{s}\right)\right]\left[X\left(\text{s’}\right)-\bar{X}\left(\text{s’}\right)\right]}$$

expresses relevant correlations and dependencies between pairs of points in *X(**s**)*, where $\rho =|s^{\prime }-s|$ denotes the spatial lag.

The BME approach categorizes all prior information known about the rate field into two major knowledge bases (KB): the general KB G and the specificatory (or site-specific) KB S, where the total knowledge base K=G∪S. As discussed in Choi et al. (2003), the G-KB is considered 'general' in the sense that it characterizes global characteristics of the rate field, such as its mean trend, spatial moments, relevant epidemiologic laws or theories, and other assumptions about the behavior of the field that may apply. On the other hand, the S-KB refers to information that is 'specific' to each mapping situation, such as observed rate values measured at specific data points. In general, the vector of random variables ***x***_*map *_representing the rate field *X(**s**) *consists of the vector of hard data random variables ***x***_*hard *_representing the latent disease rate at all locations where exact measurement values could be obtained, the vector of soft data random variables ***x***_*soft *_representing the latent rate at all locations with uncertain measured values (expressed in terms of confidence intervals or probabilistic functions), and the unknown latent rate value *x_k _*to be estimated at some estimation point, such that ***x***_*map *_= (***x***_*hard*_, ***x***_*soft*_, *x*_*k*_).

Processing the information known about the rate field consists of three main stages (Gesink Law et al., 2006), as follows (Christakos and Li, 1998; Choi et al., 2003):

(i) *Structural (or prior) stage*: The general G-KB of the rate field is considered at all mapping points corresponding to ***x***_*map*_. The structural probabilistic density function (pdf) of the random variables $x_{map,}f_{G}\left(\chi_{map}\right)$, where the vector of values ***χ***_*map *_is a realization of the random variables ***x***_*map*_, is derived by selecting the $f_{G}\left(\chi_{map}\right)$ that maximizes entropy for the given general knowledge base. Using the Shannon measure of information we have

(A3) $$\begin{array}{ccc} Info[x_{map}] & = & \log(1/f_{X}(x_{map}))\\ & = & -\log f_{X}(x_{map}), \end{array},$$

and the entropy is defined in terms of the corresponding expected information, i.e.

(A4) $$\begin{array}{c} H(x_{map})=E[Info[x_{map}]]\\ =-\int_{-\infty }^{\infty }d\chi_{map}f_{G}(\chi_{map})\log f_{G}(\chi_{map}) \end{array}$$

where *H(**x**_map_) *is the Entropy function, and E[.] is the stochastic expectation operator. Information is processed by selecting the $f_{G}\left(\chi_{map}\right)$ that maximizes *H(**x**_map_) *for the given G-KB.

(ii) *Specificatory (or meta-prior) stage*: The specificatory *S*-KB is identified and expressed in terms of hard and soft data, and the estimation point at which the field is to be estimated is defined. Here, we consider the specific framework in which all observed rates in the map are soft data ***χ***_*soft *_with measures of uncertainty due to sampling variability. In processing the data, 6 may be rewritten as

(A5) $$\chi_{soft}:\mathrm{Pr}\left[R\left(s\right)-\frac{0.5}{N\left(s\right)}\leq X\left(s\right)\leq R\left(s\right)+\frac{0.5}{N\left(s\right)}\right]=1$$

where Pr[.] is the probability operator, *R(**s**) *is the observed rate, *N(**s**) *is the population size, and *X(**s**) *is the latent rate, and ***s ***represents each region *i *where these soft data are available. Furthermore, the aim is to provide a more accurate estimate of the latent rate field only at points ***s ***where soft data is available. In other words, a soft datum expressed by A5 is available for each estimation point.

(iii) *Integration stage*: The integration (posterior) pdf, $f_{K}$, is derived by means of an operational Bayesian conditionalization rule that considers the total KB, K=G∪S, such that

(A6) $$f_{K}\left(\chi_{k}\right)=A^{-1}\overset{\infty }{\underset{-\infty }{\int }}d\chi_{soft}f_{S}\left(\chi_{k}\chi_{soft}\right)f_{G}\left(\chi_{map}\right)$$

where $\text{A}=\overset{\infty }{\underset{-\infty }{\int }}d\chi_{k}\overset{\infty }{\underset{-\infty }{\int }}d\chi_{soft}f_{S}\left(\chi_{k}\chi_{soft}\right)f_{G}\left(\chi_{map}\right)$ is the normalization parameter, and $f_{S}\left(\chi_{k}\chi_{soft}\right)$ is the multivariate uniform pdf defined by A5 jointly for the estimation point and the soft data points in its neighborhood.. The integration pdf provides a full stochastic assessment of the value *x_k _*of the latent rate field at any estimation point, from which one may derive a variety of estimators. In this work, the expected value of the integration pdf was used as an appropriate estimator${\hat{X}}_{k}$ of the latent rate, i.e.

(A7) $${\hat{X}}_{k}=\text{E}\left[x_{k}\right]=\overset{\infty }{\underset{-\infty }{\int }}d\chi_{k}f_{K}\left(\chi_{k}\right),$$

which we refer to as the BME mean estimator. Other estimators include the mode or the median of the integration pdf. Additionally, the variance of the integration pdf, or BME variance, provides a useful measure of estimation uncertainty.
